# Physical activity for the prevention and treatment of major chronic disease: an overview of systematic reviews

**DOI:** 10.1186/2046-4053-2-56

**Published:** 2013-07-10

**Authors:** David Nunan, Kamal R Mahtani, Nia Roberts, Carl Heneghan

**Affiliations:** 1 Department Primary Care Health Sciences, Radcliffe Observatory Quarter, University of Oxford, Oxford OX2 6GG, UK; 2 Bodleian Health Care Libraries, Knowledge Centre, University of Oxford, Old Road Campus Research Building, Oxford OX3 7DQ, UK

**Keywords:** Physical activity, Inactivity, Exercise, Adherence, Non-communicable disease, Management, Systematic overview

## Abstract

**Background:**

The evidence that higher levels of physical activity and/or lower levels of physical inactivity are associated with beneficial health-related outcomes stems mainly from observational studies. Findings from these studies often differ from randomised controlled trials and systematic reviews currently demonstrate mixed results, due partly to heterogeneity in physical activity interventions, methodologies used and populations studied. As a result, translation into clinical practice has been difficult. It is therefore essential that an overview is carried out to compare and contrast systematic reviews, and to identify those physical activity interventions that are the most effective in preventing and/or treating major chronic disease. This protocol has been registered on PROSPERO 2013: CRD42013003523.

**Methods:**

We will carry out an overview of Cochrane systematic reviews. We will search the Cochrane Database of Systematic Reviews for systematic reviews of randomised controlled trials that have a primary focus on disease-related outcomes. We will restrict reviews to those in selected major chronic diseases. Two authors will independently screen search outputs, select studies, extract data and assess the quality of included reviews using the assessment of multiple systematic reviews tool; all discrepancies will be resolved by discussing and reaching a consensus, or by arbitration with a third author. The data extraction form will summarise key information from each review, including details of the population(s) (for example, disease condition), the context (for example, prevention, treatment or management), the participants, the intervention(s), the comparison(s) and the outcomes. The primary outcomes of interest are the prevention of chronic disease and/or improved outcomes, in the treatment or management of chronic disease. These outcomes will be summarised and presented for individual chronic diseases (for example, any change in blood pressure in hypertension or glucose control in diabetes). Secondary outcomes of interest are to describe the structure and delivery of physical activity interventions across chronic disease conditions and adverse events associated with physical activity.

**Discussion:**

We anticipate that our results could inform researchers, guideline groups and policymakers of the most efficacious physical activity interventions in preventing and/or managing major chronic disease.

## Background

The World Health Organization (WHO) reported that physical inactivity is the fourth leading risk factor for global mortality, accounting for 6% of deaths globally [1]. A more recent analysis of the worldwide burden of disease further estimated that physical inactivity was responsible for 6% of the incidence of coronary heart disease, 7% of type 2 diabetes, 10% of breast cancer, and 10% of colon cancer. The study went on to conclude that if physical inactivity decreased by 25% then more than 1.3 million deaths could be averted every year [2].

As a result, both the UK government and relevant professional bodies recommend that health professionals take a more active role in encouraging patients to lead healthier lifestyles, including offering advice on smoking, alcohol consumption, diet and physical activity/exercise at every patient contact [3,4]. It was further hoped that the recent London Olympic Games could have been be used to improve understanding of the benefits of exercise and physical activity in the prevention and treatment of chronic conditions [5]. However, the evidence for a legacy of an automatic increase in physical activity is mixed [6].

Nevertheless, the WHO recommends that all adults should aim for 30 minutes of moderate activity daily on at least five days over a one week period, with slight variants of these recommendations for children (under 5s and 5 to 18 years) and older adults (aged 65+) [1]. These recommendations have been adopted in the UK, Europe and North America [7-9].

Data to support these recommendations were predominately sourced from epidemiological and observational studies providing evidence that higher levels of physical activity and/or lower levels of physical inactivity are associated with beneficial health-related outcomes across a wide range of populations [10-18]. These findings have been used to inform a large number of randomised controlled trials (RCTs) assessing the preventative, treatment or management benefits of physical activity and exercise interventions in specific populations, particularly those at risk of developing or already diagnosed with chronic disease [19-22]. Systematic reviews of these RCTs have been conducted to appraise, summarise and collate them (for example, the Cochrane Database of Systematic Reviews). Systematic reviews demonstrate mixed results, with some reviews of RCTs showing a benefit from physical activity whilst others do not find any benefit from physical activity for different chronic diseases [23-27]. For example, a 2004 Cochrane review of eight RCTs assessing the effects of short- and long-term, land- and water-based aerobic and aerobic with strength exercise programmes found there was no increase in pain or decrease in functional capacity but there was little improvement in aerobic capacity or muscle strength [24].

Conversely, a 2008 Cochrane review assessing the role of exercise, dietary or exercise plus dietary interventions for preventing type 2 diabetes mellitus reported that exercise only interventions were no better than usual care or diet alone interventions. Moreover, no studies have reported relevant data for diabetes- and cardiovascular-related morbidity, mortality or quality of life [23]. Decision-makers are therefore faced with a plethora of reviews that contain data of varying quality and scope, since some similar chronic disease conditions have been reviewed more than once.

### Objectives of this overview

We will carry out an overview of existing Cochrane systematic reviews. Overviews are suggested as a logical and appropriate step for comparing and contrasting separate reviews and are particularly useful where there is ambiguity, which could be removed by systematic appraisal and summarising [28]. Using this methodology our objective is to summarise the current best evidence for the use of physical activity in the prevention and/or treatment of selected chronic diseases and discuss how our findings can be used to guide future policymakers and guideline author.

## Methods

### Protocol and registration

Our protocol is registered on PROSPERO 2013 (CRD42013003523) [29].

### Data sources and search strategy

Two authors (NR and DN) devised the search strategy of the Cochrane Database of Systematic Reviews (Cochrane Library, Wiley) based on a combination of free-text keywords and subject headings that describe exercise and the disease categories of interest. Date and language restrictions will be determined as per the criteria for individual reviews. Table 1 shows the search strategy we will use to identify reviews. Since Cochrane reviews strive for methodological rigour and are regularly updated, we do not propose to include non-Cochrane reviews within this overview.

**Table 1:** Cochrane Library search strategy

***Id***	***Criteria***
*#1*	*MeSH descriptor: [Exercise] explode all trees*
*#2*	*MeSH descriptor: [Exercise Therapy] explode all trees*
*#3*	*MeSH descriptor: [Motor Activity] explode all trees*
*#4*	*MeSH descriptor: [Sports] explode all trees*
*#5*	*MeSH descriptor: [Recreation] explode all trees*
*#6*	*MeSH descriptor: [Dancing] explode all trees*
*#7*	*“exercise”:ti,ab,kw*
*#8*	*“physical activity” or “motor activity”:ti,ab,kw*
*#9*	*((physical or resistance or strength) near training):ti,ab,kw*
*#10*	*training:ti*
*#11*	sport*:ti,ab,kw
*#12*	walk or walking or bicycle* or cycling or run or jog or running or jogging:ti,ab,kw
*#13*	athlet* or basketball or cricket or dance or dancing or football* or hockey or netball or rugby or ski or skiing or soccer or swim* or volleyball:ti,ab,kw
*#14*	*“physical education” or “physical training” or “physical fitness”:ti,ab,kw*
*#15*	*#1 or #2 or #3 or #4 or #5 or #6 or #7 or #8 or #9 or #10 or #11 or #12 or #13 or #14*
*#16*	*MeSH descriptor: [Cardiovascular Diseases] explode all trees*
*#17*	*MeSH descriptor: [Heart Diseases] explode all trees*
*#18*	*MeSH descriptor: [Vascular Diseases] explode all trees*
*#19*	*MeSH descriptor: [Diabetes Mellitus] explode all trees*
*#20*	*MeSH descriptor: [Renal Insufficiency, Chronic] explode all trees*
*#21*	*MeSH descriptor: [Neoplasms by Site] explode all trees*
*#22*	*MeSH descriptor: [Neoplasms] explode all trees*
*#23*	*MeSH descriptor: [Lung Diseases, Obstructive] explode all trees*
*#24*	*MeSH descriptor: [Arthritis, Rheumatoid] explode all trees*
*#25*	*MeSH descriptor: [Osteoporosis] explode all trees*
*#26*	*cardiovascular or cardio-vascular or cvd:ti*
*#27*	((cardiovascular or cardio-vascular) near disease*):ti,ab,kw
*#28*	((cardiovascular or cardio-vascular) near (event* or outcome* or risk*)):ti,ab,kw (Word variations have been searched)
*#29*	*coronary or heart or chd or myocardial:ti (Word variations have been searched)*
*#30*	((coronary or heart) near disease*):ti,ab,kw
*#31*	((coronary or heart) near (event* or outcome* or risk*)):ti,ab,kw
*#32*	*(ischaemic or ischemic or ischaemia or ischemia):ti (Word variations have been searched)*
*#33*	((ischaemic or ischemic or ischaemia or ischemia) near disease*):ti,ab,kw
*#34*	((ischaemic or ischemic or ischaemia or ischemia) near (event* or outcome* or risk*)):ti,ab,kw
*#35*	myocardial infarct*:ti,ab,kw
*#36*	*vascular:ti (Word variations have been searched)*
*#37*	vascular near disease*:ti,ab,kw
*#38*	(vascular near (event* or outcome* or risk*)):ti,ab,kw
*#39*	*stroke:ti,ab,kw*
*#40*	atherosclero* or arteriosclero*:ti,ab,kw
*#41*	*“peripheral artery” or “peripheral arterial”:ti,ab,kw*
*#42*	*“heart failure”:ti,ab,kw*
*#43*	thrombo* or embolism or embolus or PE or DVT:ti,ab,kw
*#44*	hypertens* or anti-hypertens* or antihypertens*:ti,ab,kw
*#45*	diabet*:ti,ab,kw
*#46*	rheumat* or arthritis:ti,ab,kw
*#47*	*osteoporosis:ti,ab,kw*
*#48*	*MeSH descriptor: [Overweight] explode all trees*
*#49*	obes* or overweight:ti,ab,kw
*#50*	neoplas* or cancer* or carcinoma* or tumor* or tumour*:ti,ab,kw
*#51*	((kidney or renal) near (disease* or failure or insufficiency)):ti,ab,kw
*#52*	*MeSH descriptor: [Hypertension] explode all trees*
*#53*	*MeSH descriptor: [Blood Pressure] explode all trees*
*#54*	*MeSH descriptor: [Mental Disorders] this term only*
*#55*	*MeSH descriptor: [Depressive Disorder] explode all trees*
*#56*	*MeSH descriptor: [Depression] explode all trees*
*#57*	depress*:ti,ab,kw (Word variations have been searched)
*#58*	((mental* or mood) near/3 (ill* or disorder* or health)):ti (Word variations have been searched)
*#59*	*MeSH descriptor: [Dementia] explode all trees*
*#60*	dementia* or alzheimer*:ti,ab,kw (Word variations have been searched)
*#61*	*#16 or #17 or #18 or #19 or #20 or #21 or #22 or #23 or #24 or #25 or #26 or #27 or #28 or #29 or #30 or #31 or #32 or #33 or #34 or #35 or #36 or #37 or #38 or #39 or #40 or #41 or #42 or #43 or #44 or #45 or #46 or #47 or #48 or #49 or #50 or #51 or #52 or #53 or #54 or #55 or #56 or #57 or #58 or #59 or #60*
*#62*	*#15 and #61*
*#63*	*MeSH descriptor: [Heart Diseases] explode all trees and with qualifiers: [Rehabilitation - RH]*
*#64*	*MeSH descriptor: [Vascular Diseases] explode all trees and with qualifiers: [Rehabilitation - RH]*
*#65*	*#62 or #63 or #64*

MeSH, medical subject heading.

### Selection of reviews

Two authors (DN and KRM) will independently review the results of the above search strategy and select reviews that meet the inclusion criteria (as outlined below). Any disagreement will be resolved by discussion with a third author (CH). References will be managed using Endnote v5.0 (Thomson Reuters).

### Types of reviews

We will include any review of randomised controlled trials that examine the effects of a physical activity intervention in the prevention or management of a chronic disease (see Types of participants) and that have a primary focus on disease-related outcomes.

### Types of participants

Participants will be adults at risk of developing and/or already diagnosed with major chronic disease(s) as defined by the reviews. We will restrict reviews to those relating to the four areas highlighted in the 2008–2013 WHO non-communicable disease action plan [30]: (1) cardiovascular disease including cerebrovascular disease, heart disease (including congenital heart disease, coronary heart/artery disease (atherosclerosis), heart failure from any aetiology, heart valve disease, hypertension, ischaemic heart disease), peripheral arterial disease, deep vein thrombosis and pulmonary embolism; (2) cancer (for the purpose of this review we will limit this to breast and prostate cancer); (3) chronic respiratory disease including asthma and chronic obstructive pulmonary disease and (4) diabetes. We will also include chronic kidney disease, obesity, osteoporosis arthritis (including rheumatoid and psoriatic arthritis), depression and dementia. Since the overview is based on Cochrane Reviews we will be led by their inclusion criteria and discuss the limitations of the inclusion criteria accordingly.

### Types of intervention

We will consider all types of interventions or combinations of interventions involving physical activity, exercise, rehabilitation (involving physical activity and/or exercise) and sport in the prevention or treatment of selected major chronic diseases. These interventions can be compared with either control interventions (such as standard or usual care) or with another intervention aimed at preventing or treating major chronic disease (for example, diet for overweight or obese people).

### Types of outcomes

#### Primary outcomes

The primary outcomes of interest are the prevention of chronic disease and improved outcomes, treatment or management of chronic disease. These outcomes will be summarised and presented for individual chronic diseases; for example the incidence of diabetes, reduced mortality in coronary heart disease, the lowering of blood pressure in hypertension, better glucose control in diabetes or improved symptoms of depression.

#### Secondary outcomes

The secondary outcomes are: the structure and delivery of physical activity interventions across chronic disease conditions; adverse events (such as injury related to physical activity, exercise, rehabilitation or sports); withdrawals due to the physical activity intervention; and adherence to the intervention(s), particularly the fidelity and drop-out rate over time and the relation between drop-out and mode, intensity and volume of physical activity. Where possible, we will also report on the cost effectiveness of physical activity interventions.

### Data extraction and analysis

The methodology for data extraction and analysis will be based on the guidance in the *Cochrane Handbook of Systematic Reviews of Interventions*[31]. The full text of selected reviews will be obtained. Two authors (DN and KRM) will independently extract descriptive and outcome data from each included review using a predefined data extraction form, resolving any discrepancies by discussion and consensus; failing which, a third author (CH) will arbitrate. The data extraction form will summarise key information from each review, including details of the population(s) (for example, disease condition), the context (for example, prevention, treatment or management), the participants, the intervention(s) including method of delivery and duration, the comparison(s), outcomes and duration of follow up. Outcomes wherever possible will include both beneficial and harmful effects of the intervention(s). These data will be presented in a series of summary tables and figures.

### Assessment of methodological quality of included reviews

Two reviewers (DN and KRM) will independently assess the methodological quality of included studies using the Assessment of Multiple Systematic Reviews (AMSTAR) tool [28,32] with adapted items from the Grading of Recommendations Assessment, Development and Evaluation (GRADE). We will not exclude reviews based on AMSTAR, but will conduct sensitivity analysis if applicable to explore the consequences of synthesising reviews of differing quality. We will summarise the quality of the evidence in included reviews based on the risk of bias and any summary of findings tables within them [27].

### Dealing with missing data

We will extract reasons for missing data as reported by the original reviews. We will report the number of studies that perform intention-to-treat or per protocol analyses if provided by the original reviews.

### Data synthesis

We will present data as a narrative synthesis supported by tabulated data of the statistical outcomes reported in the original reviews. Comparisons presented will be determined by the data in the original reviews. Where possible we will group data by chronic disease condition and whether reviews focused on prevention or treatment and management. We aim to ensure in all cases that we compare interventions that reflect clinical decisions.

The outcomes of interest will be either dichotomous or continuous. We will report risk ratios and their corresponding 95% confidence intervals and *P* values for dichotomous outcomes, and mean differences for continuous outcomes. Flow diagrams will be used to summarise the study selection process. We will use summary tables and figures to present results in a structured and clear format, which will enhance the textual commentary. We will also use the Preferred Reporting Items for Systematic Reviews and Meta-Analyses (PRISMA) statement [33] as a framework to guide our selection of items for our systematic review of reviews.

The reporting of outcomes without quantitative data will be descriptive. Lastly, we will provide a list of excluded studies with reasons for exclusion.

### Sensitivity analysis

We will conduct sensitivity analysis based on GRADE level, by comparing results from all studies against those excluding low-quality studies.

### Sub-group analysis

We will classify the reviews into sub-groups based on the type of intervention (prevention or treatment/management), setting (for example, primary care, secondary care or community based) and type of physical activity (for example, structured or unstructured, aerobic or resistance and supervised or unsupervised) to aid interpretation and to impose a clinically relevant structure (for example, age over 75s and disease severity, where this is reported in the original review).

### Reliability of the outcomes

Where reviews have pooled outcomes (that is, meta-analyses), the heterogeneity of the evidence for each primary outcome will be examined by summarising the range of the *I*^2^ statistic. Where reviews have not pooled outcomes, we will critically evaluate the role of the outcome measure by comparing the sensitivity and stability of measures across the included reviews where possible.

### Summary of the evidence base

We will analyse and discuss limitations in the evidence base including overall methodological quality of the individual systematic reviews and the number of participants within each review. This will include an analysis of the strongest and weakest evidence for each chronic disease condition and intervention, taking into account the strength of the summary results. We will also analyse and discuss barriers to physical activity interventions and implementation in clinical practice.

## Discussion

### Expected significance of the study

The findings of this overview of reviews will have implications for policy, practice and research. Our results will provide clarity about the best evidence available on physical activity in the prevention and/or treatment of major chronic disease compared to usual care, placebo or no intervention. They will inform clinicians and policymakers of the disease conditions for which physical activity intervention provides a clear prevention and/or management benefit, and those for which there is no clear benefit or where there is a lack of evidence of benefit. If sufficient data can be extracted, the findings should also indicate the type, intensity, volume, duration and setting (that is, primary care, secondary care or community based) of physical activity interventions that result in improved health status but also the conditions under which physical activity may cause harm (that is, injury).

The overview will identify specific considerations that would need to be taken into account for future studies, such as study location; content of physical activity interventions (for example, type, intensity and duration); behavioural aspects; measurement of physical activity level; duration of study and sample size that would result in the maximal patient benefit. Finally, we hope to identify the major barriers to physical activity interventions, particularly with reference to primary care settings.

## Abbreviations

AMSTAR: Assessment of Multiple Systematic Reviews; GRADE: Grading of Recommendations Assessment Development and Evaluation; NHS: National Health Service; NIHR: National Institute for Health Research; PRISMA: Preferred Reporting Items for Systematic Reviews and Meta-Analyses; RCT: Randomised controlled trial; MeSH: Medical subject heading; WHO: World Health Organization.

## Competing interest

The authors declare that they have no competing interests.

## Authors’ contributions

DN conceived the review. DN and KRM designed the overview, and will be involved in data acquisition. NR informed the search strategy and will perform the search. DN will analyse the data with input from KRM and CH; the same authors will participate in the interpretation of the results. All authors were involved in the drafting of this protocol. All authors read and approved the final manuscript.
